# Deep learning-based electroencephalic decoding of the phase-lagged transcranial alternating current stimulation

**DOI:** 10.3389/fnhum.2025.1545726

**Published:** 2025-06-20

**Authors:** Jeongwook Kwon, Byoung-Kyong Min

**Affiliations:** ^1^Department of Brain and Cognitive Engineering, Korea University, Seoul, Republic of Korea; ^2^Institute of Brain and Cognitive Engineering, Korea University, Seoul, Republic of Korea

**Keywords:** brain stimulation, cognitive system, deep-learning, electroencephalography, top-down processing, transcranial alternating current stimulation

## Abstract

We investigated whether the phase-lag types of cross-frequency coupled alternating current stimulation (CFC-tACS), a non-invasive technique aimed at enhancing cognitive functions, could be decoded using task-based electroencephalographic (EEG) signals. EEG recordings were obtained from 21 healthy individuals engaged in a modified Sternberg task. CFC-tACS was administered online for 6 s during the middle of the retention period with either a 45° or 180° phase lag between the central executive network and the default mode network. To decode different phase-lag tACS conditions, we trained a modified EEGNet using task-based EEG signals before and after the online tACS application. When utilizing parietal EEG signals, the model achieved a decoding accuracy of 81.73%. Feature maps predominantly displayed EEG beta activity in the parietal region, suggesting that the model heavily weighted the beta band, indicative of top-down cognitive control influenced by tACS phase-lag type. Thus, EEG signals can decode online stimulation types, and task-related EEG spectral characteristics may indicate neuromodulatory activity during brain stimulation. This study could advance communicative strategies in brain–machine interfacing (BMI)-neuromodulation within a closed-loop system.

## 1 Introduction

Memory is a crucial aspect of human cognition, and numerous researchers have explored its neurophysiological bases from various perspectives. In particular, working memory is integral to a myriad of everyday cognitive tasks and is essential for activities such as problem-solving, learning, and communication. It involves the temporary storage and manipulation of information (Cowan, 2014). Essentially, two key mechanisms are associated with working memory: one for retaining information and the other for voluntary or executive control (Miller et al., 2018). Working memory processing is not confined to single brain structures; rather, it involves large, distributed networks that span different cortical areas within the cortex (Christophel et al., 2017) and extend between cortical and subcortical areas (Spellman et al., 2015). For instance, the dorsolateral prefrontal cortex (DLPFC), which involves the functional antagonism of the default mode network (DMN) (Menon, 2011; Murphy et al., 2020; Sridharan et al., 2008) and corresponds to the central executive network (CEN), plays a role in monitoring and manipulating information (Barbey et al., 2013). Neurophysiological evidence indicates that working memory is also associated with specific neuronal oscillations (Roux and Uhlhaas, 2014; Sauseng et al., 2010; Wilsch and Obleser, 2016). Additionally, previous studies have noted that the retention period of working memory is characterized by cross-frequency coupling, where the frequencies or powers of different frequencies interact (Abubaker et al., 2021; Axmacher et al., 2010).

Considerable research has focused on directly modulating neurophysiological phenomena in the brain to enhance cognitive functions or treat cognitive disorders (Liu et al., 2022). Brain stimulation techniques are categorized into invasive and non-invasive methods. Invasive stimulation, involving the implantation of a device in a targeted brain area, provides a direct and rapid neuromodulatory effect. However, the costs and surgical requirements of invasive neuromodulation limit its use among healthy individuals. Conversely, non-invasive neuromodulation involves stimulation applied through the scalp without the need for surgery (Bikson et al., 2019). Among non-invasive methods, transcranial current stimulation (tCS) has gained popularity due to its adaptability, portability, and relatively lower cost. In particular, transcranial alternating current stimulation (tACS) has proven effective in neurophysiologically modulating human cognitive functions by synchronizing brain waves through the application of electrical stimulation analogous to endogenous brain waves (Herrmann et al., 2013). It also modulates brain rhythms, enhances activity in specific frequency bands, and can be applied locally.

In this study, we employed theta/alpha-gamma phase-amplitude coupled tACS, drawing on evidence linking the theta-gamma ratio with short-term memory capacity and theta/alpha-gamma phase-amplitude coupling with working memory (Abubaker et al., 2021; Kim et al., 2022). Phase differences in stimulation have been shown to affect performance (Polanía et al., 2015). For instance, in-phase stimuli (e.g., a relative phase difference of 0°) enhance functional coupling between distant regions, while out-of-phase stimuli (a relative phase difference of 180°) reduce phase matching and impair performance on cognitive tasks (Polanía et al., 2012; Violante et al., 2017). Accordingly, we administered two different stimulus phase-lag conditions while participants engaged in a modified Sternberg task, a well-established working memory task (Sternberg, 1966). Stimuli were presented as a partially in-phase tACS condition when the relative phase difference was 45°, and as an out-of-phase tACS condition when the relative phase difference was 180° across CEN and DMN.

Moreover, this study explored whether brain-stimulation parameters could be decoded using task-based electroencephalographic (EEG) signals. EEG-based brain-machine (or brain-computer) interfacing (BMI or BCI) studies have historically been prominent in the field of neurotechnology, primarily focusing on using brain signals. Recently, brain-stimulation-mediated neuromodulation, which involves applying electric or magnetic energy to brain regions for intentional non-invasive neuromodulation, has garnered increasing attention. Interactive studies between BMI/BCI and neuromodulation are crucial to bridge these two uni-directional approaches, aiming to establish a communicatory method between brain-stimulation-mediated neuromodulatory techniques and EEG-based BMI/BCI technology. Specifically, if a brain-stimulation parameter can be decoded using EEG signals, this technique could facilitate the online updating of brain-stimulation parameters based on real-time EEG features, particularly within a BMI-neuromodulation closed-loop feedback system.

For this purpose, deep learning models for classification were employed. Notably, convolutional neural networks (CNNs) are popular in the classification of EEG data (Craik et al., 2019; Kim et al., 2021; Roy et al., 2019), attributed to their efficacy in various applications such as image classification (He et al., 2016) and speech recognition (Gulati et al., 2020). Owing to their superior feature extraction performance, CNNs excel in EEG data classification (Lawhern et al., 2018; Schirrmeister et al., 2017). In this study, we used EEGNet (Hong et al., 2023; Lawhern et al., 2018), a simplified CNN-based model designed for learning parameters from a small dataset while maintaining robust performance across diverse paradigms. EEGNet models are particularly advantageous for extracting interpretable neurophysiological features relevant to the task paradigm, rendering them ideal for training deep learning models using electrophysiological data from cognitive processes. Specifically, the operation of EEGNet's first layer resembles a wavelet transform to identify the focus frequency bands of the trained layer, which acts as a filter bank (Lawhern et al., 2018). Therefore, by examining the frequency bands of the trained layer, we can determine the weight of the model for a particular frequency range.

Although numerous studies have decoded EEG, few have concentrated on decoding EEG signals acquired during the tACS stimulation paradigm. By analyzing online stimulation EEG data, the effectiveness of current stimulation techniques and their neurophysiological correlates can be assessed. Therefore, we examined whether task-based EEG signals could decode the phase-lag type of cross-frequency coupling-based non-invasive alternating current stimulation (CFC-tACS) applied, specifically distinguishing between 45° and 180° phase-lag stimulation across CEN and DMN.

## 2 Materials and methods

### 2.1 Participants

Twenty-one healthy individuals (six female, mean age 24.0 ± 3.1) participated in this study. All participants had normal or corrected-to-normal vision and no history of psychiatric or neurological disorders. Each participant provided written informed consent. The study was conducted in accordance with the ethical guidelines of the Institutional Review Board of Korea University (KUIRB-2021-0209-08) and the Declaration of Helsinki (World Medical Association, 2013).

### 2.2 Materials and procedures

**Figure 1:**
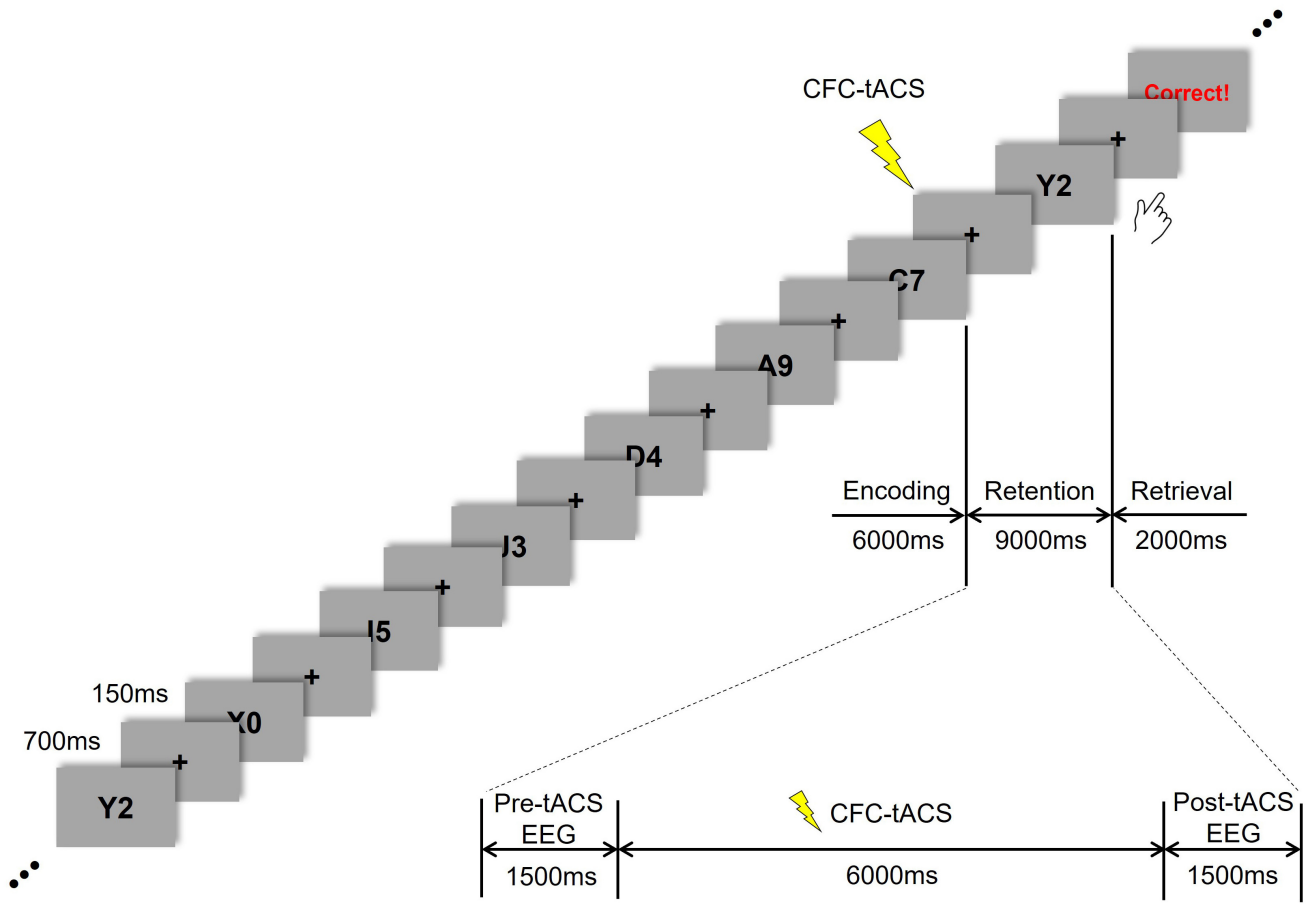
Time flow of Sternberg task and tACS treatment.

The participants were instructed to perform a modified version of the Sternberg task (Sternberg, 1966), as illustrated in Figure 1. In this task, seven stimuli consisting of combined letters and numbers were presented sequentially during the encoding period. Each stimulus appeared for 700 ms, with an inter-stimulus interval of 150 ms. This was followed by a 9 s retention period of working memory, during which CFC-tACS was applied to the participants for 6 s (from 1.5 to 7.5 s within the retention period). Subsequently, during a 2 s retrieval period, participants responded to indicate whether the item presented during the retrieval was among those shown during the encoding period. At the end of the response period, three types of feedback were provided based on the participant's response: correct, incorrect, or no response.

Seven sessions were conducted, each consisting of 30 responses. The first session involved performing the Sternberg task without tACS treatment. The second and third sessions included one phase-lag tACS treatment (either 45° or 180°). The fourth session involved performing the Sternberg task without tACS treatment. In the fifth and sixth sessions, the tACS stimulus was applied in the alternate phase-lag condition (180° or 45°) during the Sternberg task. The seventh session involved performing the Sternberg task without tACS treatment again. This study was conducted in a double-blind manner, and the order of 45° and 180° phase-lag CFC-tACS sessions was counterbalanced across participants. The task was administered using E-prime software (E-prime 3.0 Professional, Psychology Software Tools, USA). This study evaluated the EEG data from the tACS sessions 2, 3, 5, and 6.

### 2.3 EEG recordings and tACS

The 64 Ag/AgCl EEG electrodes (Brain Products GmbH, Germany) were positioned on the scalp using an EEG cap (actiCAP, Brain Products GmbH, Germany) in accordance with the international 10-10 system. EEG signals were recorded at a sampling frequency of 500 Hz using a BrainAmp DC amplifier (Brain Products GmbH, Germany). The reference electrode was placed at the tip of the nose, while the ground electrode was located in the AFz channel. EEG data were offline filtered using a band-pass infinite impulse response filter from 4 to 50 Hz. To extract peri-tACS EEG data, 500-ms segments were epoched from 1 s before (i.e., 0–0.5 s during the retention period) and 1 s after (i.e., 8.5–9 s during the retention period) the tACS treatment to avoid potential artifacts induced by tACS during EEG recording (Figure 1). A detrending procedure was applied to each epoch to eliminate DC offsets. For artifact rejection, epochs were removed if the peak amplitude exceeded ±100 μV or if the slope exceeded 50 μV/ms. In addition, EEG epochs contaminated by eye movement were rejected by manual inspection.

High-density transcranial alternating current stimulation (HD-tACS) was administered using a Soterix MxN-65 (Soterix Medical Inc., USA), with the frequency and intensity of stimulation tailored for each participant. Given the correlation between CFC of neuronal oscillations and working memory performance (Abubaker et al., 2021), and our hypothesis that the neuromodulatory effect would be maximized when the stimulus waveform closely resembles the target human brain wave, the stimulus waveform in this study was designed in a CFC manner. The amplitude and phase frequencies of the stimuli were individually calculated as follows (Kim et al., 2022).


(1) $$\begin{array}{l} Stimulus\;signal=\;\frac{{\bar{A}}_{f_{A}}}{2}(\sin(2\pi f_{p}t)+1)\sin\left(2\pi f_{A}t\right)\\ \;+\;{\bar{A}}_{f_{p}}\sin\left(2\pi f_{P}t\right) \end{array}$$

(2) $$\begin{array}{lll} Return\;signal & = & -\frac{1}{N}\left(Stimulus\;signal\right) \end{array}$$

*f*_*A*_ and *f*_*P*_ represent the amplitude and phase frequencies, respectively, and ${\bar{A}}_{f_{A}}$ and ${\bar{A}}_{f_{p}}$ are constants that determine the maximum intensity of the stimulus set to 2:8, where *N* denotes the number of return channels.

**Figure 2:**
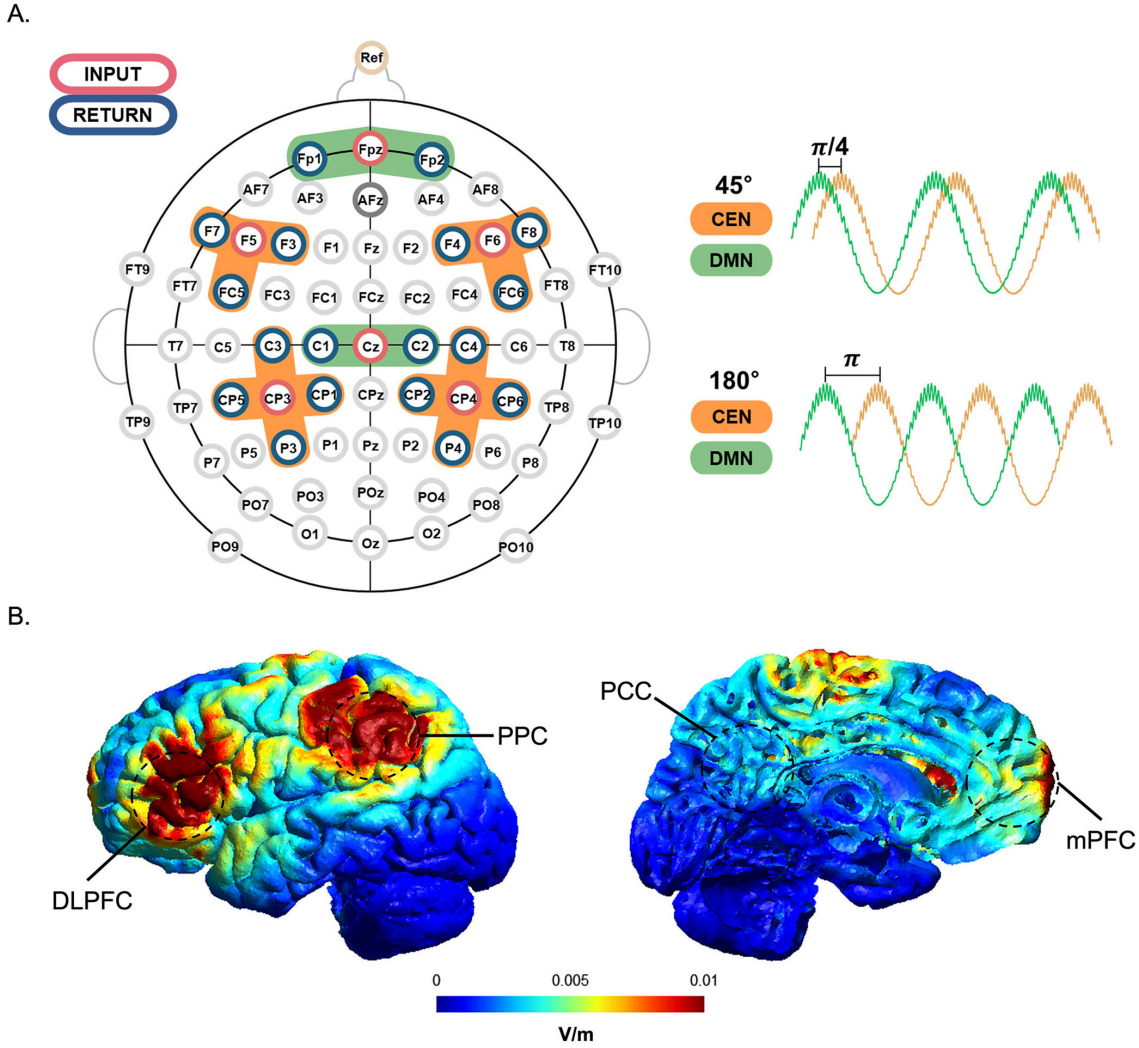
tACS channel montage and tACS simulation with a phase lag. **(A)** The stimulation input electrode for each region of interest (orange for CEN; green for DMN) is marked in red, and its surrounding return electrodes are marked in blue. Cross-frequency coupled tACS was applied with multi-electrode setups designed to modulate DMN (mPFC and PCC, time course in green) with a phase lag of 45° or 180° preceding that of CEN (DLPFC and PPC, time course in orange). **(B)** Simulation of the tACS-induced electric field in the target regions. Cortical maps (lateral and medial views) show the simulated neuromodulation of the tACS target areas. The unit *normE* denotes the normalized strength of the induced electric field (V/m), with high intensities indicated in red and low intensities represented in blue.

Previous studies have demonstrated that applying a stimulus with the same frequency and phase difference to two different areas can either promote or disrupt the synchronization of functional networks (Helfrich et al., 2014; Polanía et al., 2015; Violante et al., 2017). In this study, the stimulation targets were the CEN and the DMN, which functionally antagonize each other. The CEN is activated during cognitive tasks, while the DMN is deactivated when cognitive engagement is required. Stimulation was applied to the CEN and DMN with a phase lag of 45°, partially in-phase, and 180°, out-of-phase (Seo et al., 2025). The stimulus was delivered to these two target regions through six cortical regions: the left and right dorsolateral prefrontal cortex (DLPFC) and posterior parietal cortex (PPC) for the CEN, and the medial prefrontal cortex (mPFC) and posterior cingulate cortex (PCC) for the DMN (Figure 2). To examine whether the target regions were optimally stimulated and the stimulation signals matched the intended phase lag, simulation was performed using SimNIBS (ver. 3.2.6, DRCMR & DTU, Denmark) (Thielscher et al., 2015) and tES LAB (ver. 3.0, Neurophet, Seoul, Korea) software. We individually adjusted the stimulation intensity for each participant in a stepwise manner to ensure that it remained below the individual sensation threshold and that the total stimulation intensity did not exceed 1.5 mA.

### 2.4 Data analysis

Preprocessing of the EEG data was performed using the Brain Vision Analyzer (Brain Products GmbH, Germany), MNE Python (Gramfort et al., 2014) and Scipy (Virtanen et al., 2020) libraries. To analyze the effect of online stimulation, we compared tACS-mediated changes in the 500 ms EEG spectral power 1 s before (i.e., 0–0.5 s in the retention period) and 1 s after (8.5–9 s in the retention period) the tACS treatment (Figure 1). Consistent with previous studies on working memory that have identified task-relevant activation in the frontal midline (Gevins et al., 1997; Jensen and Tesche, 2002; Onton et al., 2005) and parietal-occipital regions (Jensen et al., 2002; Sauseng et al., 2005) during working memory tasks, we used EEG signals from the frontal (Fz, F1, and F2), parietal (Pz, P1, and P2), and occipital (Oz, O1, and O2) regions to decode the tACS phase-lag types in this study.

### 2.5 EEG decoding

**Figure 3:**
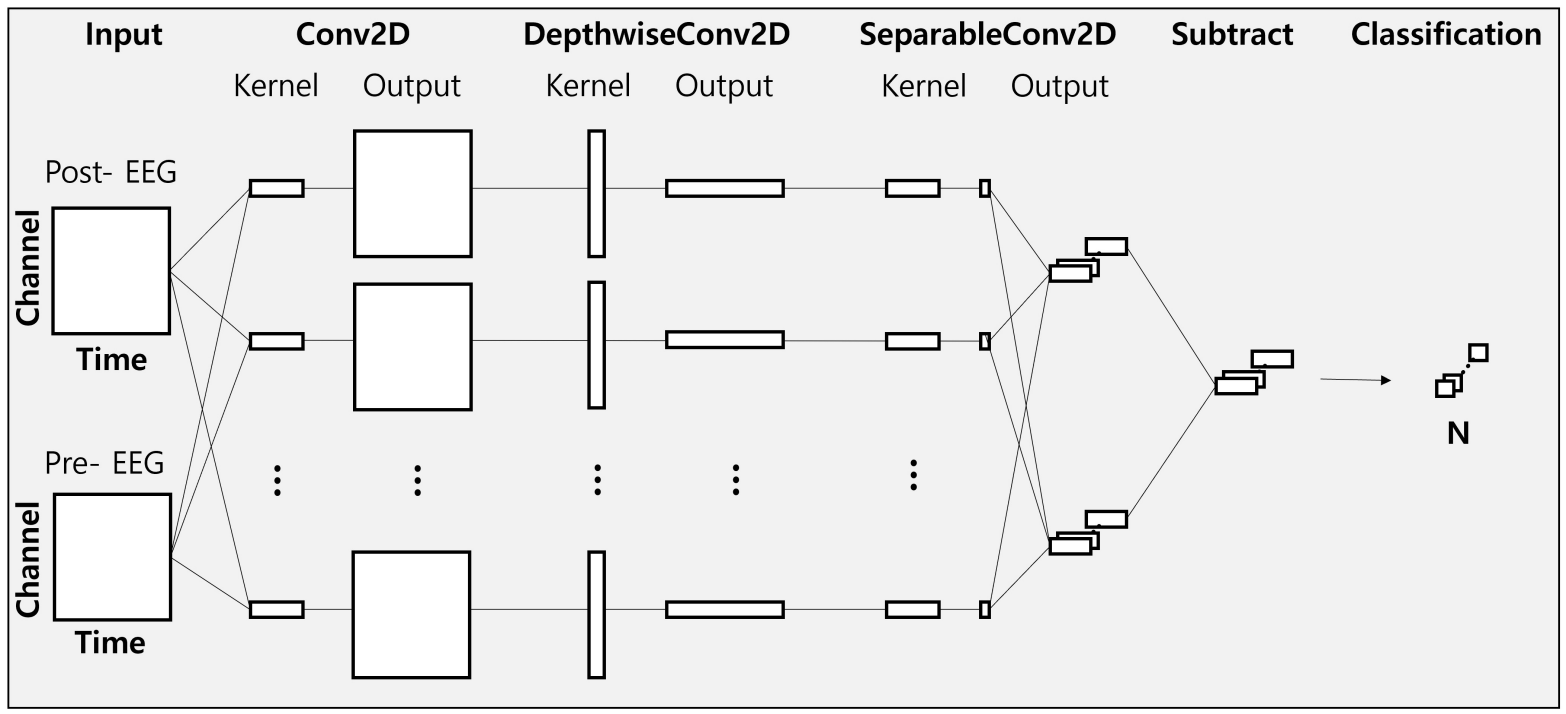
Visualization of the modified EEGNet architecture.

**Figure 4:**
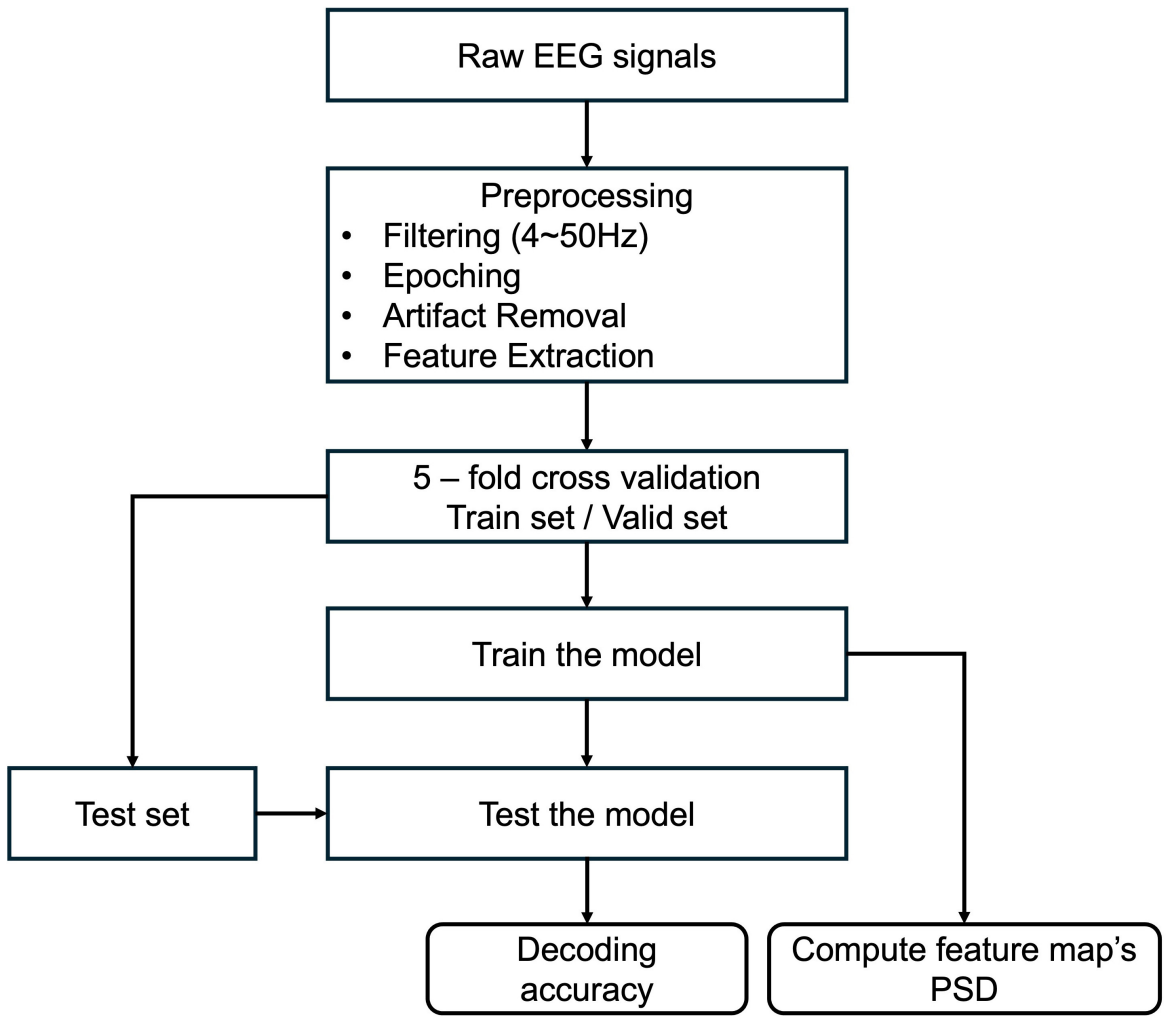
Flowchart of analysis procedures. After preprocessing the raw EEG data, a 5-fold cross-validation was conducted. Decoding accuracies were subsequently computed and compared across various classification models. Finally, power spectral density (PSD) was calculated to derive the model's feature map.

To elucidate the changes in EEG features between different phase-lag tACS conditions, we employed the EEGNet model, frequently used in deep-learning-based EEG studies (Hong et al., 2023; Kim et al., 2021; Zhu et al., 2021). We adapted the architecture of this model (hereafter referred to as modified EEGNet; Figure 3) to assess changes in the feature vectors of raw EEG data from the pre- to post-tACS periods (Figure 4). In addition, we evaluated decoding performance using feature vectors trained exclusively on data from either the pre- or post-tACS period.

The EEGNet model utilized in this study comprises three convolutional layers: a standard 2D convolution layer, a depth-wise convolution layer, and a separable convolution layer. The first convolution layer functions as a band-pass filter, the depth-wise convolution layer serves as a frequency-specific spatial filter, and the separable convolution layer aggregates temporal features. A softmax layer is subsequently used for classification.

The parameters of the EEGNet architecture (Figure 3) were set as follows: C = 9 (number of channels); T = 64 (number of time points for the pre-tACS period), T = 64 (number of time points for the post-tACS period), or T = 128 (number of time points for the entire epoch period); F1 = 8 (number of filters in the Conv2D layer); D = 2 (number of spatial filters in DepthwiseConv2D); F2 = 16 (number of filters in the SeparableConv2D); N = 2 (number of classes). For training the EEGNet model, we employed He initialization to set the model's weights and the Adam optimizer (α = 0.001, decay parameters β1 = 0.9 and β2 = 0.999) to minimize the binary cross-entropy loss function. The batch size and training iterations (epochs) were set at 16 and 300, respectively.

The EEG dataset was partitioned into training and test datasets at a 4:1 ratio. The models underwent training over 300 epochs, with parameter tuning conducted using a validation dataset through a 5-fold cross-validation (Lemm et al., 2011) to obtain the performance of out-of-sample classification; the model that performed best in validation was subsequently chosen for final classification accuracy verification on the test dataset. Decoding was conducted for each cluster to identify the features that significantly influenced model performance. Since the average computation time for EEGNet-based decoding over 500 iterations was ~0.67 ms (SD: 0.12 ms), it appears feasible for implementation in a BMI-neuromodulation closed-loop feedback system.

To ascertain which frequency-band and brain-region features were most crucial for differentiating phase-lag stimulation conditions, the model was trained on each individual feature. The EEG data were decomposed into different frequency bands, and their decoding performances were evaluated. Decoding was executed after applying bandpass filtering for the theta (4–8 Hz), alpha (8–13 Hz), beta (13–30 Hz), and gamma (30–50 Hz) bands to assess changes in decoding performance relative to using broadband (4–50 Hz) signals. Given that tACS-mediated beta activity exhibited a biphasic response, the beta band was further segmented into lower (13–20 Hz) and upper (20–30 Hz) beta bands. Due to the short epoch length (500 ms), the delta band (below 4 Hz) was omitted from the analysis.

Decoding accuracies based on regions of interest were further analyzed to determine which brain region contributed most significantly to decoding performance. For this purpose, decoding was conducted using bandpass-filtered (4–50 Hz) EEG data corresponding to each specified brain region: electrodes Fz, F1, and F2 for the frontal region; electrodes Pz, P1, and P2 for the parietal region; and electrodes Oz, O1, and O2 for the occipital region. Throughout this study, all channels refer to these nine electrodes.

The first layer of the EEGNet trains the target frequency band through convolution in the time domain. To identify the frequency bands that most accurately represent the changes in EEG spectral features between pre- and post-tACS periods, the EEG datasets from these periods were convolved using a shared weight. The power spectral density (PSD) was calculated to determine which frequency band was predominantly activated in the trained layer. To examine the model's feature map, a feature map was generated using the parietal EEG signals, which demonstrated the highest feature importance in this study. This feature map was created by calculating the PSD after the EEG signal passed through the learned filter weights of the first convolutional layer. The PSD of the filter was normalized to the maximum power value for each participant, and the feature map was normalized to the maximum power of a single trial after computing the PSD and then averaged across trials. All analyses were conducted using Python (Python Software Foundation, https://www.python.org) with the scikit-learn (Pedregosa et al., 2011) and Pytorch (Paszke et al., 2019) libraries. Decoding was performed individually for each participant.

To evaluate the decoding performance of the proposed EEGNet model against other classifiers, classification was conducted using linear discriminant analysis (LDA), support vector machine (SVM), and random forest (RF) classifiers. Each model underwent training with 5-fold cross-validation, and the best-performing model was assessed using the test dataset. Each model's training involved the use of Welch's power spectral density (Welch, 1967) to quantify the change in PSD between the pre- and post-tACS periods.

### 2.6 Statistical analysis

Although the absence of a sham condition may raise concerns about potential tACS vs. no-tACS confounds, both the 45° and 180° conditions involved active stimulation; thus, decoding was performed strictly between two phase-lagged tACS states, eliminating the possibility that results reflect general stimulation effects. False discovery rate (FDR)-corrected paired-sample *t*-tests were conducted to compare measures between the two phase-lag tACS conditions, and one-sample *t*-tests were used to determine if the decoding performance of the models significantly differed from chance level. To statistically evaluate the variables that significantly influenced decoding performance of the model, decoding was conducted for each frequency band and brain region. The results of each decoding process were compared with those of the model decoded using all channels and broadband signals to ascertain any statistical difference in performance. All analyses and statistical procedures were executed using Python (Seabold and Perktold, 2010).

## 3 Results

### 3.1 Decoding performance across models

**Figure 5:**
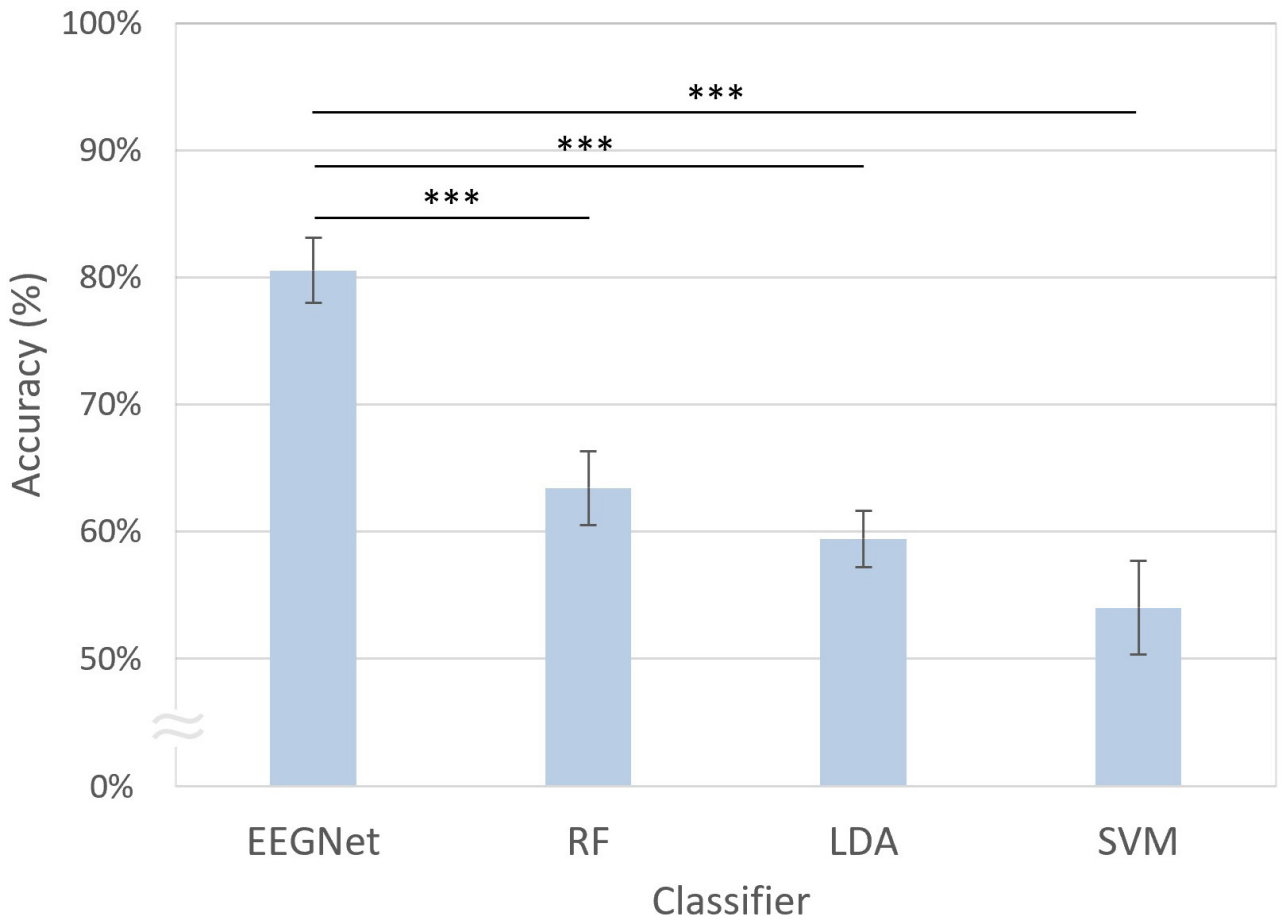
Comparison of decoding accuracies across different classifiers. The error bars represent the standard errors of the mean. RF, random forest; LDA, linear discriminant analysis; SVM, support vector machine. Asterisks indicate statistical significance (***p < 0.001, FDR-corrected).

As shown in Figure 5, the modified EEGNet exhibited significantly higher performance (80.55%) compared with other machine learning methods. Specifically, EEGNet outperformed random forest, which exhibited a decoding accuracy of 63.43% [*t*_(20)_ = 4.42, *p* < 0.001, FDR-corrected], LDA with 59.44% [*t*_(20)_ = 6.09, *p* < 0.001, FDR-corrected], and SVM which achieved 54.01% [*t*_(20)_ = 5.76, *p* < 0.001, FDR-corrected].

### 3.2 Decoding performance using pre- and post-tACS EEG signals

**Figure 6:**
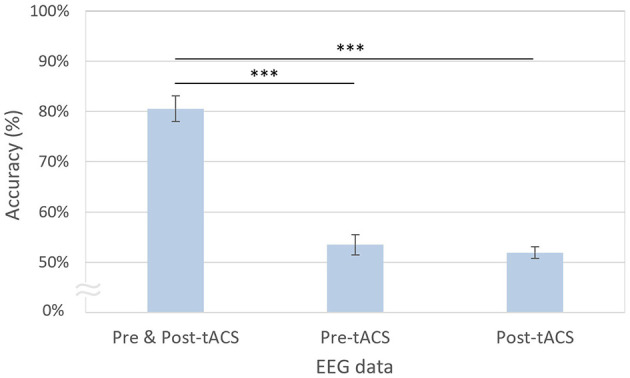
Modified EEGNet-based decoding accuracies using pre- and post-tACS periods. The error bars represent the standard errors of the mean. Asterisks indicate statistical significance (***p < 0.001, FDR-corrected).

The decoding accuracies using both pre- and post-tACS EEG signals (80.55%) were significantly higher compared with using only pre-tACS [53.48%; *t*_(20)_ = 10.57, *p* < 0.001, FDR-corrected] or only post-tACS EEG signals [51.91%; *t*_(20)_ = 8.25, *p* < 0.001, FDR-corrected; Figure 6]. However, the decoding accuracies using only the pre-tACS EEG signals [*t*_(20)_ = 1.70, *n.s*.] and the post-tACS EEG signals [*t*_(20)_ = 1.67, *n.s*.] were not significantly different from chance level.

**Figure 7:**
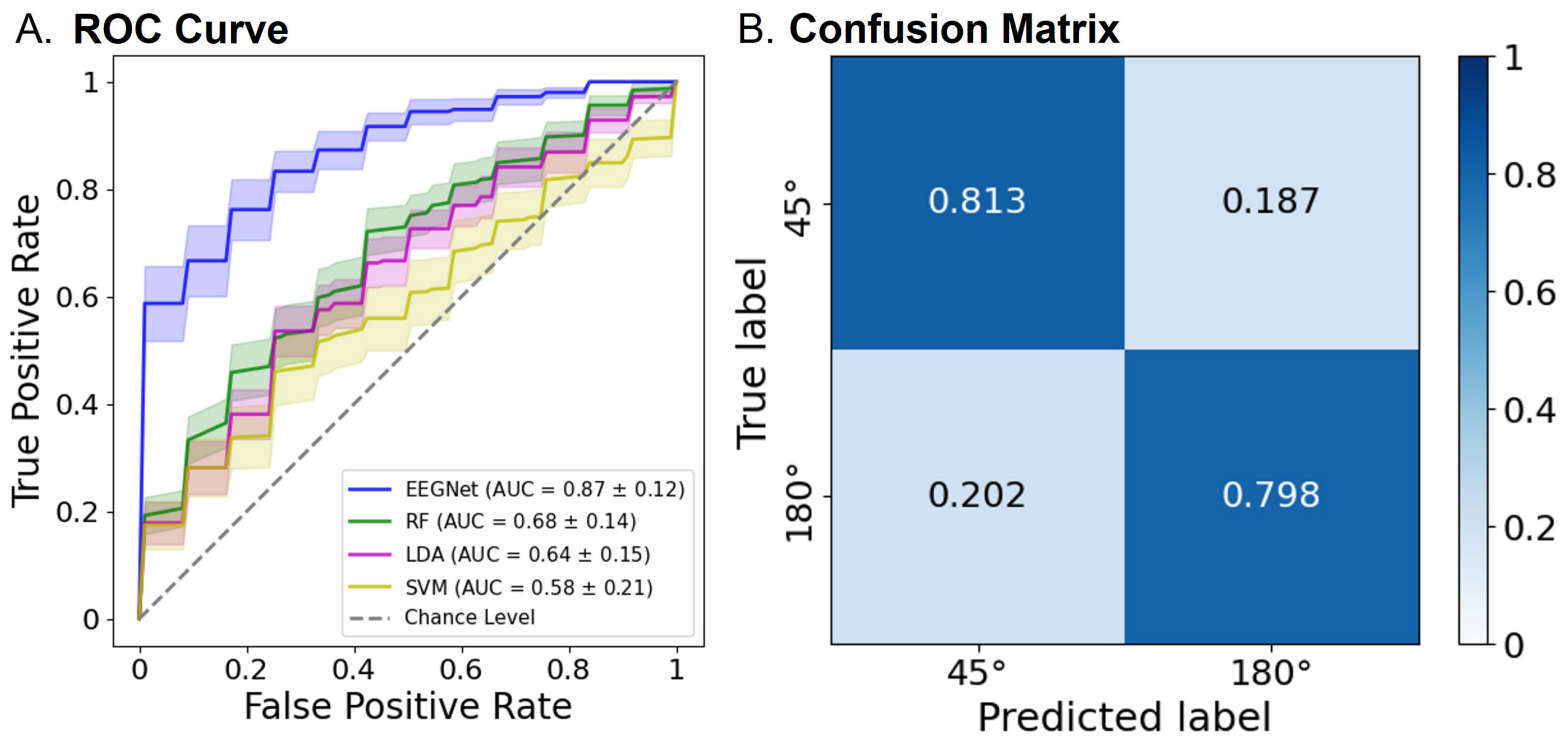
Classification performance of the EEGNet model using both pre- and post-tACS EEG signals. **(A)** The area under the curve (AUC) scores in the receiver operating characteristic (ROC) curves were obtained through 5-fold cross-validation using both pre- and post-tACS periods. Each curve represents the ROC curve for each test dataset, with the corresponding AUC scores (mean ± standard errors of the mean) noted within the legend [modified EEGNet in blue; random forest (RF) in green; LDA in pink; SVM in yellow]. The gray dotted line indicates the chance level. Error bands indicate standard errors of the mean. **(B)** Confusion matrix of the EEGNet model classification results.

Consistently, as illustrated in Figure 7, the modified EEGNet achieved significantly higher AUC scores (0.87) in the receiver operating characteristic (ROC) curves compared with other machine learning methods. Specifically, EEGNet outperformed RF with an AUC of 0.68 [*t*_(20)_ = 4.60, *p* < 0.001, FDR-corrected], LDA with an AUC of 0.64 [*t*_(20)_ = 5.29, *p* < 0.001, FDR-corrected], and SVM with an AUC of 0.58 [*t*_(20)_ = 5.46, *p* < 0.001, FDR-corrected].

### 3.3 Decoding performance based on EEG frequency bands

**Figure 8:**
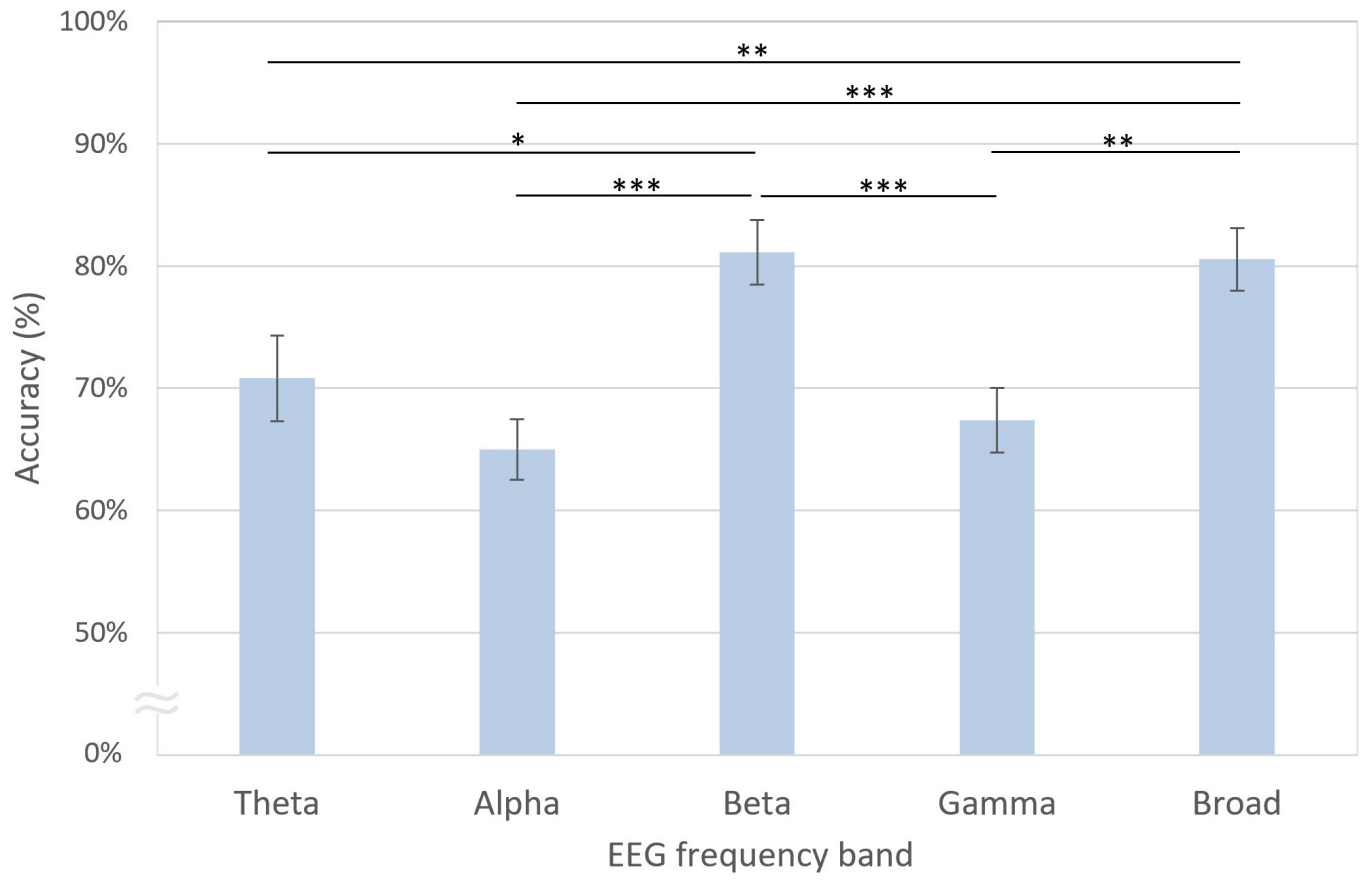
Decoding accuracies based on EEG frequency bands. The error bars represent standard errors of the mean. Asterisks indicate statistical significance (*p < 0.05, **p < 0.01, ***p < 0.001, FDR-corrected).

As shown in Figure 8, the decoding accuracy of the beta band was the highest at 81.12%, which was not significantly different from that of the broadband EEG signals at 80.55% [*t*_(20)_ = −0.24, *n.s*.]. In contrast, the accuracies of the theta [70.82%; *t*_(20)_ = 3.71, *p* < 0.01, FDR-corrected], alpha [65.01%; *t*_(20)_ = 5.66, *p* < 0.001, FDR-corrected], and gamma [67.37%; *t*_(20)_ = 3.41, *p* < 0.01, FDR-corrected] bands were significantly lower compared with the broadband. When comparing decoding accuracies across individual frequency bands, the beta band demonstrated superior decoding performance relative to the theta [*t*_(20)_ = 2.73, *p* < 0.05, FDR-corrected], alpha [*t*_(20)_ = 5.54, *p* < 0.001, FDR-corrected], and gamma bands [*t*_(20)_ = 4.64, *p* < 0.001, FDR-corrected].

### 3.4 Decoding performance based on regions of interest

**Figure 9:**
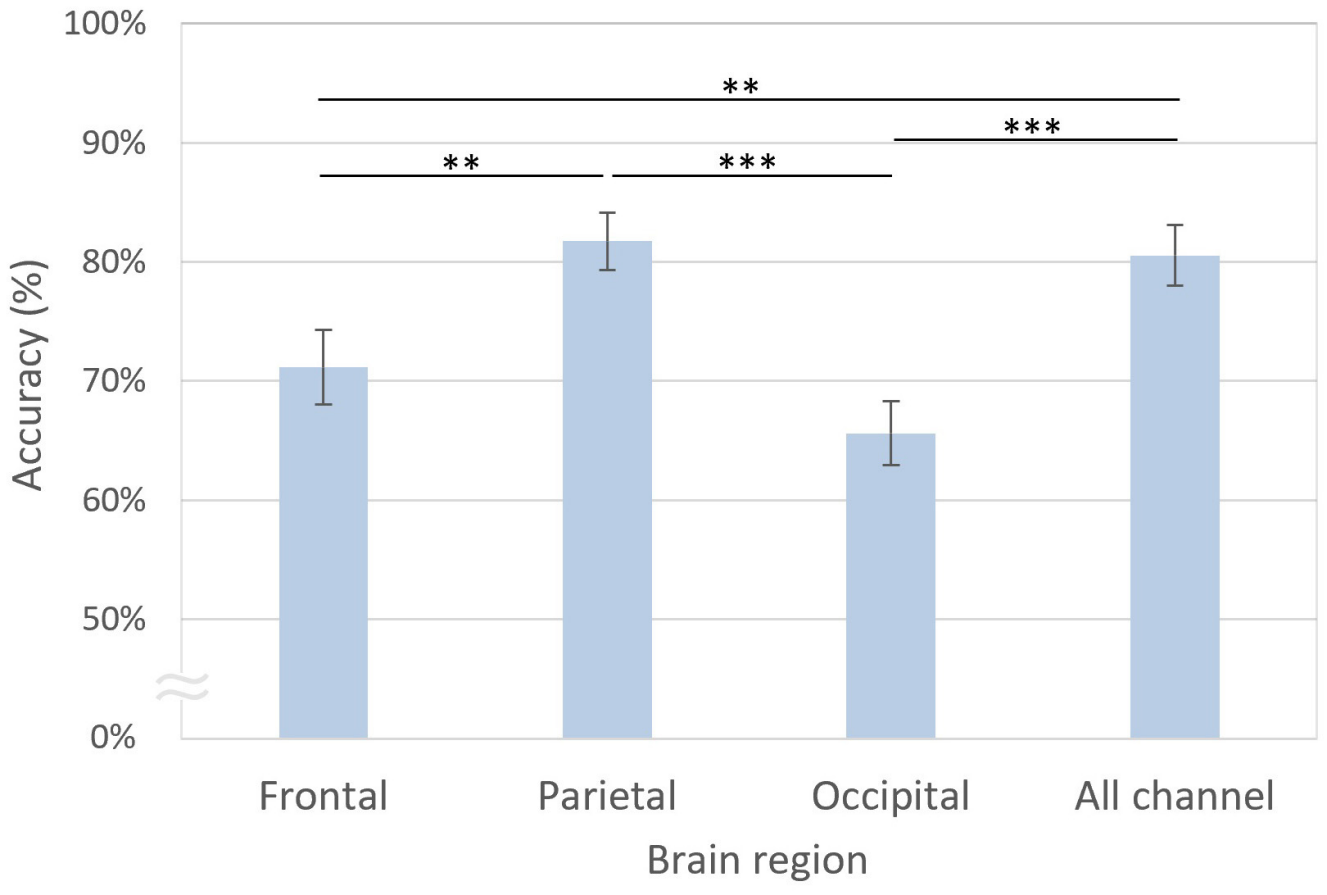
Decoding accuracies based on brain regions. The error bars represent standard errors of the mean. Asterisks indicate statistical significance (**p < 0.01, ***p < 0.001, FDR-corrected).

The decoding accuracy based on the parietal channels was the highest at 81.73%, which was not significantly different from that of all channels at 80.55% [*t*_(20)_ = 0.60, *n.s*.; Figure 9]. However, the decoding accuracies of the frontal [71.16%, *t*_(20)_ = 3.08, *p* < 0.01, FDR-corrected] and occipital [65.62%, *t*_(20)_ = 5.27, *p* < 0.001, FDR-corrected] regions were significantly lower than those of the parietal region. Similarly, the decoding accuracies of the frontal [*t*_(20)_ = 3.70, *p* < 0.01, FDR-corrected] and occipital [*t*_(20)_ = 5.77, *p* < 0.001, FDR-corrected] regions were significantly lower than those using all channels.

**Figure 10:**
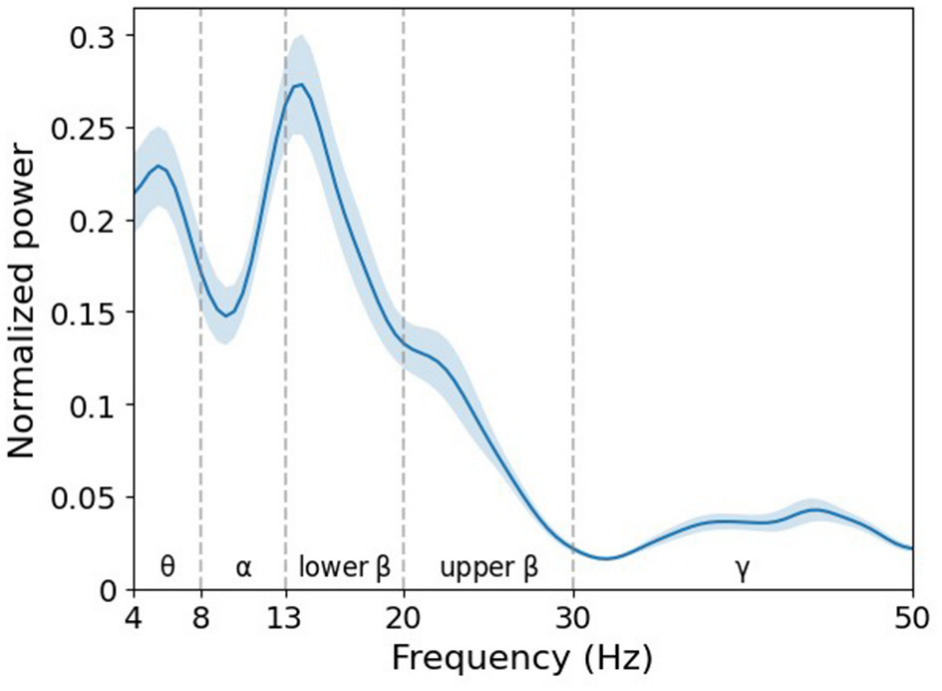
Grand average of normalized power spectral density (PSD) of filter weight. The learned filter weights of the first convolutional layer of the EEGNet model trained on broadband data were projected to the frequency domain via fast Fourier transform. Note that the decisive spectral features in the decoding model are detected in the lower beta band. Error bands indicate standard errors of the mean.

**Figure 11:**
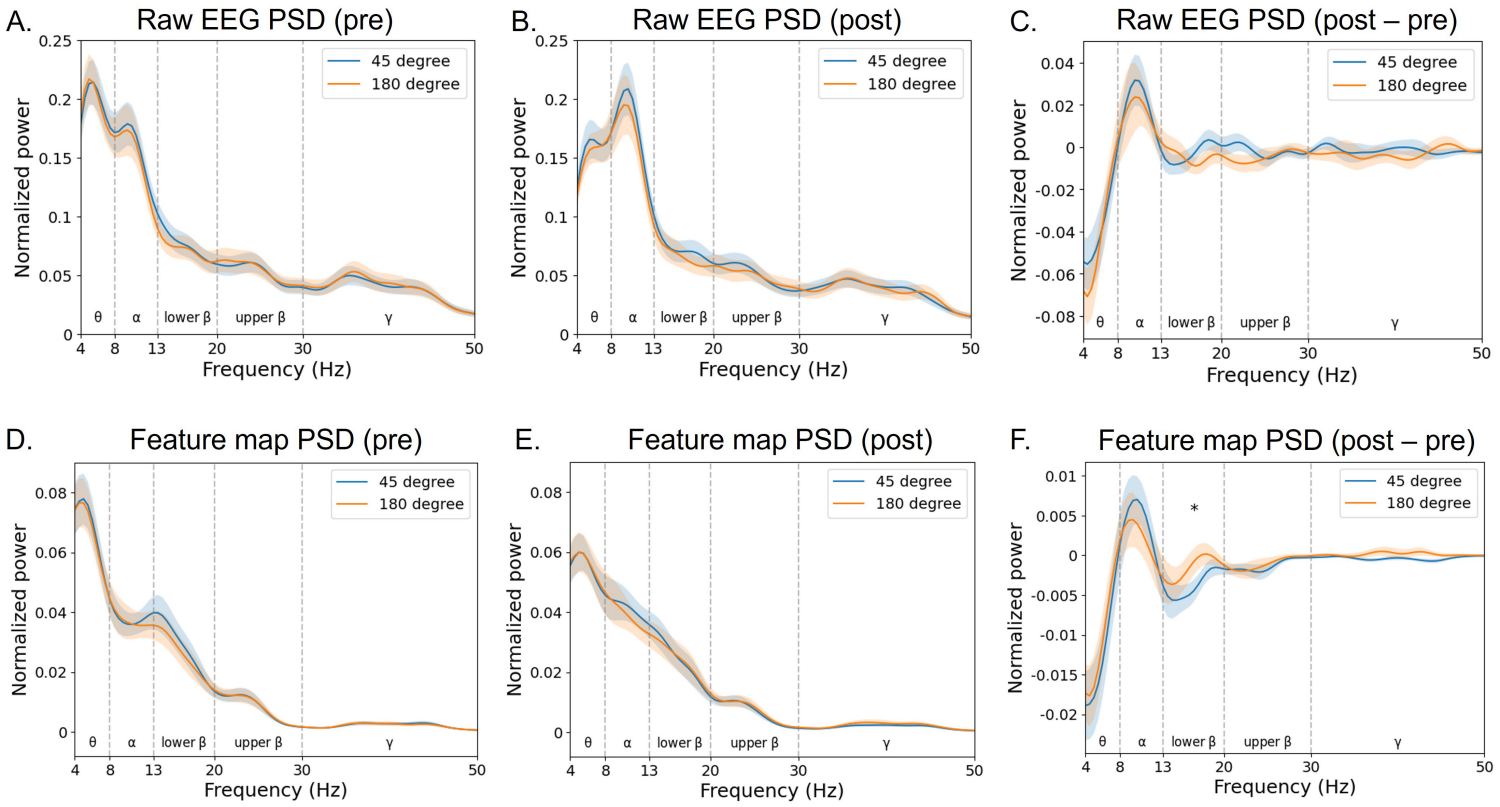
Grand average of normalized power spectral density (PSD) of the feature map. The PSD of input raw EEG signals **(A–C)** and corresponding feature maps **(D–F)** of the EEGNet model trained on broadband data were projected to the frequency domain via fast Fourier transform (at the parietal region averaged across electrodes Pz, P1, and P2). The blue curves indicate the 45 phase-lag tACS, whereas the red ones indicate the 180 phase-lag tACS. The notion “pre” indicates the pre-tACS period **(A, D)**, and “post” indicates the post-tACS period **(B, E)**. The notion “post–pre” indicates post-tACS minus pre-tACS PSD of raw EEG or feature data **(C, F)**. Error bands indicate standard errors of the mean. An asterisk indicates statistical significance (*p < 0.05).

In particular, the trained filter exhibited a peak in the lower beta band (Figure 10). In the feature map (Figure 11), the difference in normalized PSD of the parietal region between the pre- and post-tACS periods was consistently dominant in the lower beta band [*t*_(20)_ = −2.18, *p* < 0.05].

## 4 Discussion

In the present study, we performed decoding of task-based EEG signals to determine whether two phase-lags of CFC-tACS could be differentiated at the single-trial EEG level. Further analysis revealed that the dominant features for decoding were associated with EEG beta activity and parietal regional activation (Figures 8, 9). To assess the decoding performance of the modified EEGNet compared with other classification models, we utilized conventional machine learning techniques such as SVM or LDA. The modified EEGNet demonstrated superior performance, particularly in decoding the differences in spectral and spatial features between the pre- and post-tACS EEG signals compared with using either pre- or post-tACS EEG signals alone. Given that previous studies have indicated that tACS during a working memory task influences not only EEG power but also functional connectivity (Abellaneda-Pérez et al., 2020; Kim et al., 2022), incorporating functional connectivity as an additional tACS-mediated feature may enhance the decoding performance of stimulation parameters in future studies.

Importantly, the study of the convolutional layer filter weights in the EEGNet model revealed interpretable neurophysiological correlates of phase-dependent tACS treatment, with EEG beta band activity in the parietal region being the most discriminative feature critically contributing to classification performance. The first layer of EEGNet is a convolutional layer that trains a temporal filter to identify which frequency bands exhibit differences (Lawhern et al., 2018). To identify a common pattern in the trained models, we calculated the PSD of the convolutional layer for each participant and found that the dominant filter was formed in the EEG beta band (Figure 10). Consistently, the feature map showed pronounced differences between pre- and post-tACS in the lower beta band (Figure 11F).

Several studies have highlighted two main roles of beta activity in working memory function (Miller et al., 2018). One role involves the inhibition and removal of information held in working memory (Lundqvist et al., 2018; Schmidt et al., 2019), and the other pertains to the maintenance of working memory (Gelastopoulos et al., 2019; Kopell et al., 2011; Salazar et al., 2012). In cognitive tasks, the beta band is instrumental in top-down processing (Richter et al., 2017), including selective attention (Lee et al., 2013; Palacios-García et al., 2021; Richter et al., 2017). In the context of working memory, top-down modulation and selective attention are crucial mechanisms for the encoding, maintenance, and retrieval of working memory (Gazzaley and Nobre, 2012). Furthermore, beta oscillations in working memory have been linked to the maintenance of the current brain state and the (re)activation of currently task-relevant contents (Engel and Fries, 2010; Spitzer and Haegens, 2017). Collectively, these findings highlight the significant association of beta activity with working memory function.

Moreover, EEG signals from the parietal region significantly contributed to the decoding performance (Figure 9). Specifically, parietal beta activity is known to be crucial for the maintenance of working memory, while prefrontal beta activity is associated with the inhibition of working memory (Deiber et al., 2007; Gelastopoulos et al., 2019; Kopell et al., 2011; Lundqvist et al., 2018; Miller et al., 2018; Salazar et al., 2012; Schmidt et al., 2019). Given that our EEG data were collected during the maintenance period of the working memory task, and the inhibition process in the prefrontal region primarily occurs after retrieval, our findings concerning parietal beta activity align with previous research. Additionally, parietal lower beta activity, which demonstrated significant differences in normalized PSD between the two phase-lag stimulation conditions in the feature map, has been noted for its potential role as a dynamic buffer in working memory that can be modulated by top-down processing (Gelastopoulos et al., 2019). This suggests that the modulation of top-down regulation, such as selective attention or working memory, might be feasible by manipulating the tACS phase difference across the CEN and DMN.

The present study demonstrated that EEGNet can automatically identify robust task-relevant EEG features, facilitating practical and ubiquitous EEG-based applications for decoding types of neuromodulatory stimuli on state-of-the-art platforms. As larger datasets are progressively accumulated for observations dependent on neuromodulatory parameters, our deep-learning approach could further uncover more stable and generalizable features corresponding to various neuromodulatory spectrums. This advancement could enable the accurate classification and stratification of neuromodulatory stimulation types, enhancing the precision and effectiveness of neurostimulation therapies. Nonetheless, the current decoding approach holds potential for further improvement in future studies. A key limitation lies in the relatively small sample size, which may have constrained the statistical power of our results. Therefore, this limitation should be considered when interpreting the findings. To address the challenge of limited EEG data, several data augmentation strategies—such as generative adversarial networks (Haradal et al., 2018; Ramponi et al., 2018) or random transformations (e.g., rotation, jittering, scaling, and frequency warping) (Freer and Yang, 2020)—may help generate sufficient data volumes, facilitating the application of deep learning techniques.

In summary, the present study demonstrated the feasibility of decoding a stimulation parameter using task-based EEG signals, indicating that task-relevant EEG signals can reflect the neural signatures of specific brain stimulation types. Notably, this study decoded the phase-lag type of stimulation, which is a more complex parameter compared to stimulation intensity or frequency. In future research, this approach could contribute to the development of a dynamic closed-loop EEG-tACS system. For instance, based on the decoding results of ongoing EEG signals, brain-stimulation parameters could be updated in real-time to enhance human cognitive functions. In this regard, the present study could pave the way for innovative communication methods in an interactive BMI/BCI-neuromodulation closed-loop platform.

## Data Availability

The raw data supporting the conclusions of this article will be made available by the authors, without undue reservation.
